# Supplementary material for: Supercooled erythritol for high-performance seasonal thermal energy storage
Source: Nat Commun. 2024 Jun 11;15:4948. doi: 10.1038/s41467-024-49333-7 (PMC11166931; doi:10.1038/s41467-024-49333-7)
Supplement: Supplementary file 1 — Supporting Information [file 41467_2024_49333_MOESM1_ESM.pdf]

Supporting Information

Title

Supercooled erythritol for high-performance seasonal thermal energy storage

Author list

Sheng Yang[1,2], Hong-Yi Shi[1,2], Jia Liu[1,2], Yang-Yan Lai[1,2], Özgür Bayer[3], Li-Wu Fan[1,2]🗁

Affiliations

[1]*State Key Laboratory of Clean Energy Utilization, Zhejiang University, Hangzhou 310027, People's Republic of China*

[2]*Institute of Thermal Science and Power Systems, School of Energy Engineering, Zhejiang University, Hangzhou 310027, People's Republic of China*

[3]*Department of Mechanical Engineering, Middle East Technical University, 06800, Ankara, Türkiye*

🗁Corresponding author. Tel./fax: +86 571 87952378.

E-mail address: liwufan@zju.edu.cn (L.-W. Fan).

SI 1. Preparation and characterization of the food-thickener-enhanced erythritol

1.1. Flowchart of sample preparation and characterization

The flowchart of preparation and characterization of the thickened-erythritol samples by the three gum-type food thickeners is shown in Supplementary Figure 1. In addition to the chemical and thermal characterizations, some samples that cannot crystallize spontaneously were tested for triggering of crystallization. Details regarding the active triggering tests are discussed in SI 5.

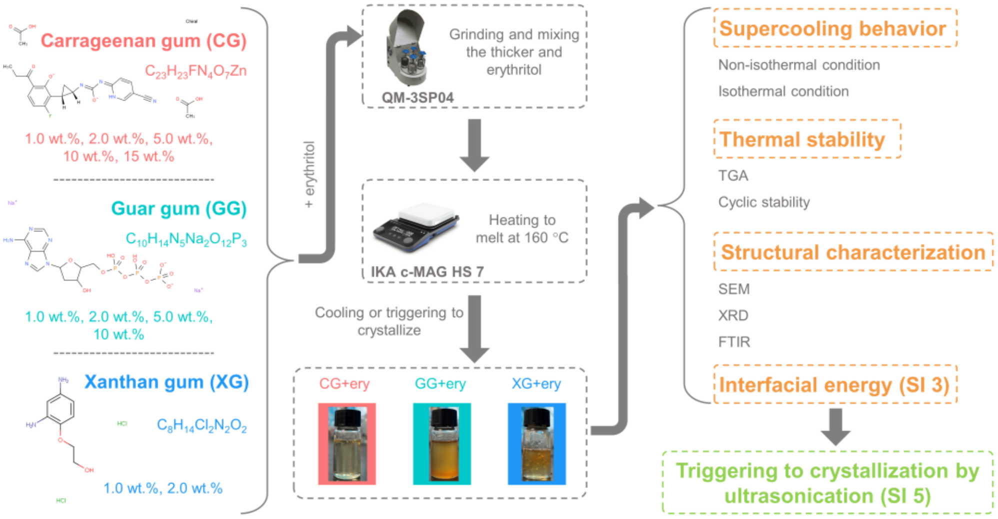

Supplementary Figure 1. The flowchart for preparation and characterization of the thickened erythritol.

1.2. Setup for the isothermal heating/cooling test

The setup for the isothermal heating/cooling cycle test is depicted in Supplementary Figure 2, where the main unit is the insulation chamber for holding the samples.

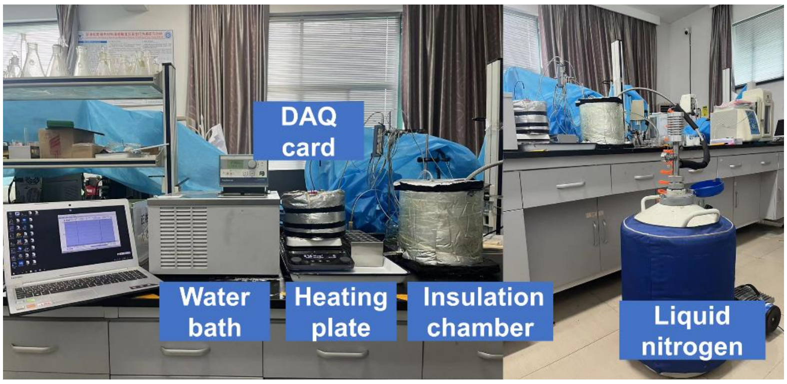

Supplementary Figure 2. The experimental setup for testing the isothermal heating/cooling performance.

1.3. Determination on the rheological behaviors

The cone-plate type rheometer (Anton Paar, MCR102) used is shown in Supplementary Figure 3.

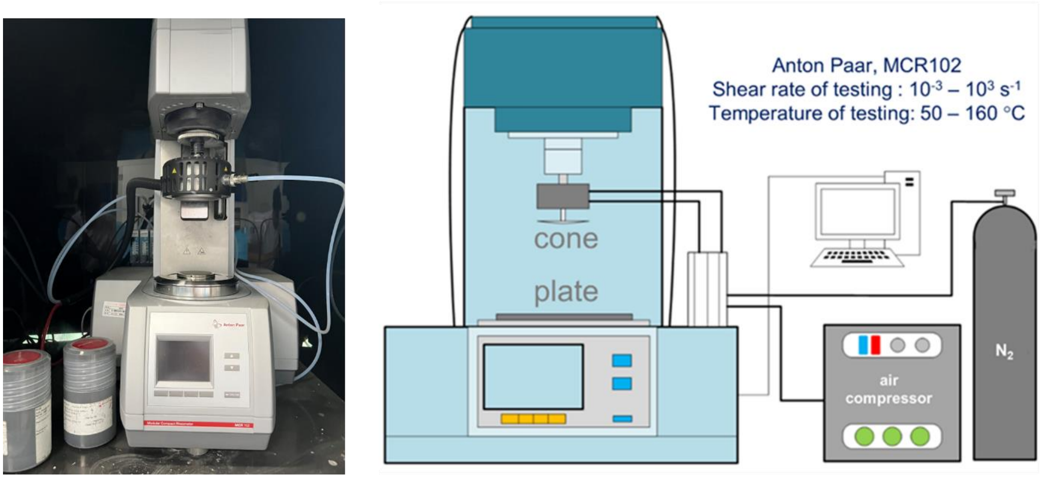

Supplementary Figure 3. The photograph and schematic diagram of the rheometer.

1.4. Thermophysical properties of erythritol and CG-thickened erythritol

The major thermophysical properties of pure erythritol and two CG-thickened erythritol samples at high loadings are listed in Supplementary Table S1, where the melting point (T_m), latent heat of fusion (ΔH_m) and special heat capacity (C_p) were measured by differential scanning calorimetry (DSC, NETZSCH DSC 200 F3), and the density was measured by a densitometer (Anton Paar, DMA5000M).

Supplementary Table 1 The major thermophysical properties of erythritol and CG-thickened erythritol at high loadings.

	pure erythritol	10 wt.%	15 wt.%
T_m (°C)	~118.1±0.7	107.0±1.3	104.7±2.2
ρ (solid at 20°C) (kg·m^{-3})	1412±39	1384±27	1361±13
ρ (liquid at 150°C) (kg·m^{-3})	1338±14	1301±8	1250±36
$C_{p,s}$ (solid at 20°C) (J·g^{-1}K^{-1})	1.38±0.11	1.32±0.09	1.30±0.13
$C_{p,l}$ (liquid at 120°C) (J·g^{-1}K^{-1})	2.97±0.17	2.84±0.24	2.74±0.11
ΔH_m (J·g^{-1})	335.2±2.2	279.1±1.1	259.3±15.5

1.5. Effect of cold crystallization on the latent heat of crystallization

As discussed in the main text (with Fig. 3), the latent heat of crystallization exhibits an unusual variation for the CG-thickened erythritol at high loadings (> 10 wt.%), i.e., the latent heat of crystallization is entirely composed of the latent heat released during cold crystallization upon the subsequent heating process, rather than the cooling process. Therefore, as the temperature for cold crystallization of the heavily-loaded samples was observed to keep increasing upon consecutive charging/discharging cycles, the measured latent heat of crystallization (by cold crystallization) also varies with each cycle.

In order to provide a solid thermodynamic elucidation on this observation, here we would like to start from the basic calculation of the latent heat of a PCM. In general, the latent heat refers to the difference in enthalpy before and after the phase transition of a material, such as melting or crystallization. Since the nominal melting point of a pure substance is fixed, it might give people the impression that the latent heat is a constant value, which is not always true.

During the charging process, the latent heat of fusion ΔH_{m} can be estimated by

$$\Delta H_{\mathrm{m}} = H_{T1} - H_{T2} \tag{S1-1}$$

where H_{Ti} is the enthalpy of the PCM at the temperature of Ti, e.g., $T1$ is the temperature at the end of the melting process, and $T2$ is the temperature at the beginning of the melting process. The PCM is in solid phase when the temperature is lower than $T2$, and in liquid phase when temperature is higher than $T1$.

Similarly, during the charging process, the latent heat of crystallization ΔH_c can be given by

$$\Delta H_{\mathrm{c}} = H_{T3} - H_{T4} \tag{S1-2}$$

where $T3$ is the temperature at the beginning of the crystallization process, $T4$ is the temperature at the end of the crystallization process.

Due to the high degree of supercooling of erythritol, and the even higher degrees of supercooling of the CG-thickened erythritol, the temperature of cold crystallization T_{cc} becomes much lower than the melting point T_{m}. As shown in Supplementary Figure 4a, the specific heat capacity value of erythritol in the supercooled liquid state is up to 2.6 $\mathrm{J \cdot g^{-1}K^{-1}}$, while in the solid state it is less than 1.6 $\mathrm{J \cdot g^{-1}K^{-1}}$. Such a marked difference of the specific heat

capacity between the solid and supercooled liquid phases, at the same temperature, leads to an unusual observation that the latent heat of crystallization, which is totally from cold crystallization, of the CG-thickened erythritol at high loadings increases with raising the temperature of cold crystallization. The difference in the latent heat discharged at different temperature can be intuitively expressed by the shaded area in Supplementary Figure 4a.

This reasoning can be better elucidated as the *T*-*H* diagram depicted in Supplementary Figure 4b. At the melting temperature, the solid erythritol, or any other highly supercooled PCM, absorbs heat and melts into a molten liquid. During the discharging process, the molten erythritol enters a supercooled state upon being cooled down, due to the stable supercooling behavior. Assuming two cold crystallization temperatures of $T_{cc,1}$ and $T_{cc,2}$, with $T_{cc,1}$ being the lower one, the $\Delta H_{c,1}$ is clearly less than $\Delta H_{c,2}$ because the enthalpy of the supercooled liquid phase decreases at a higher rate than that of the solid phase due to their difference in specific heat capacity.

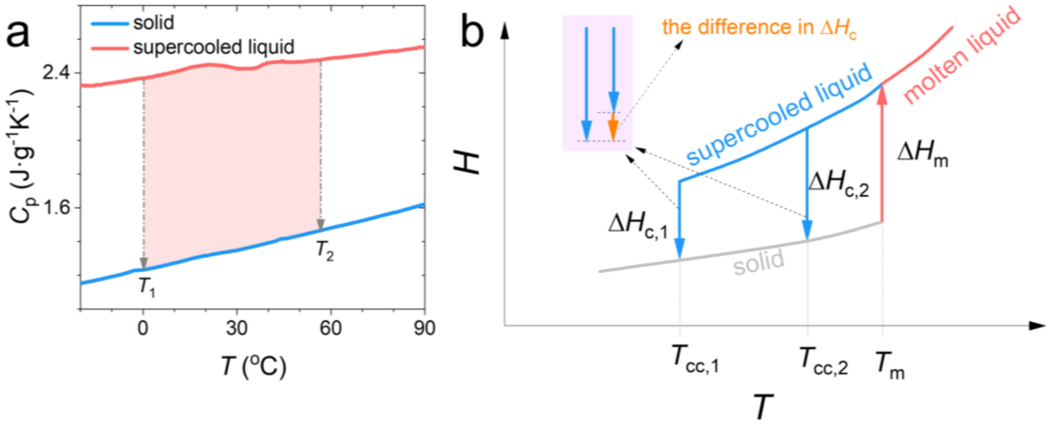

Supplementary Figure 4 The specific heat capacity and enthalpy of supercooled PCM. **a** The temperature-dependent specific heat capacity of erythritol in both solid and supercooled liquid phases. **b** The change of enthalpy upon the transition of melting and crystallization.

Therefore, the ΔH_c of the 15 wt.% CG thickened-erythritol can even become greater than that of the 10 wt.% sample, due to increasing the temperature of cold crystallization temperature, as shown in Supplementary Table 2.

Supplementary Table 2 The temperature of cold crystallization (T_{cc}) and latent heat of crystallization (ΔH_c) of CG-thickened erythritol at high loadings.

Number of cycles	10 wt.%		15 wt.%	
	T_{cc} (°C)	ΔH_c (J·g)	T_{cc} (°C)	ΔH_c (J·g^{-1})
1	−3.7	138.8	4.8	118.8
2	−2.0	144.9	2.7	115.0
3	19.2	155.1	5.2	122.2
4	21.4	156.3	10.9	135.7
5	23.0	154.8	18.9	155.8
6	23.6	152.0	23.6	156.6
7	23.6	157.2	28.0	158.9
8	24.2	158.3	31.6	161.3
9	24.0	156.6	31.7	158.9
10	23.9	153.1	32.1	162.3
11	23.2	154.0	30.7	167.9
12	23.5	156.0	31.6	161.2
13	23.5	153.8	32.5	160.7
14	23.8	152.7	32.8	157.1
15	23.8	152.2	33.0	162.0

In addition, cold crystallization is a common phenomenon observed in sugar alcohols, like in polymers. It occurs when the temperature decreases to a point where the intrinsic power of crystallization potential of the sugar alcohol becomes insufficient to facilitate molecular diffusion and crystallization. This crystallization process can only occur when the temperature rises, rather than being cooled down. For the highly thickened-erythritol (10 wt.% and 15 wt.%), the increasing in viscosity with the number of heating/cooling cycles leads to the increase of the temperature of cold crystallization, as shown in Supplementary Table 2.

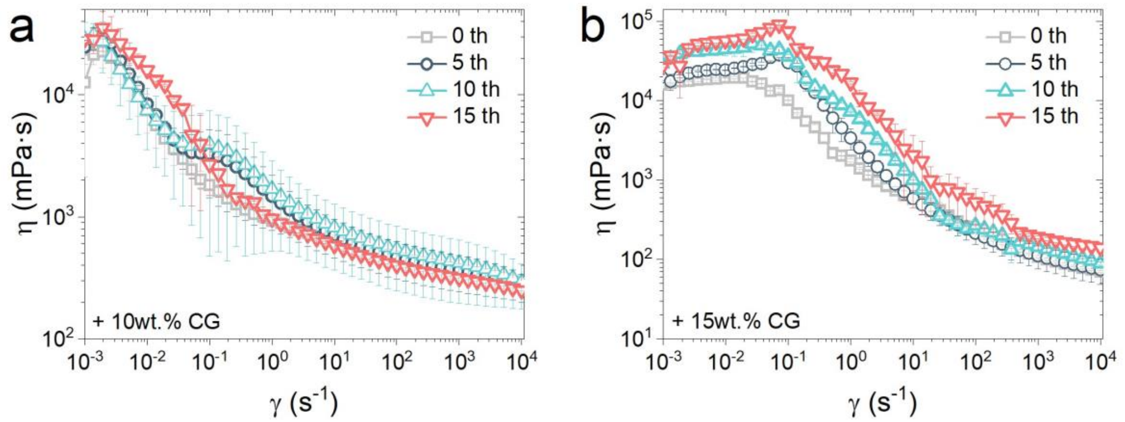

Supplementary Figure 5 The change in viscosity with the number of cycles for the case of (a) 10 wt.% CG and (b) 15 wt.% CG.

As shown in Supplementary Figure 5, the viscosity of the 15 wt.% CG-thickened erythritol become much larger with the cycling proceeds, which further intensifies the cold crystallization phenomenon. As the viscosity decreases with increasing the temperature, a higher temperature is required for lowering the diffusion resistance of the thickened erythritol molecules, thus gradually raising the onset temperature of cold crystallization. Therefore, compared to the 10 wt.% sample, the remarkable cycling-induced viscosity variation of the 15 wt.% sample leads to the gradual increase in the temperature of cold crystallization.

SI 2. Comparison of the performance among the three gum-type thickeners

2.1. Melting point and latent heat of fusion

Given in Supplementary Table 3 are the two important properties, i.e., the melting point (T_m) and latent heat of fusion (ΔH_m) of the samples, which were measured using the DSC, as mentioned in previous Section 1.2.

Supplementary Table 3 Information and properties of the raw materials and composite PCM samples.

Material/sample	Chemical formula	T_m (°C)	ΔH_m (J·g^{-1})
erythritol (ery)	$C_4H_{10}O_4$	~118.1±0.7[1–3]	335.2±2.2
carrageenan gum (CG)	$C_{23}H_{23}FN_4O_7Zn$	160.8±21.7	–
guar gum (GG)	$C_{10}H_{14}N_5Na_2O_{12}P_3$	156.9±15.4	–
xanthan gum (XG)	$(C_{35}H_{49}O_{29})_n$	170.7±30.3	–
1 wt.% CG/ery	–	116.4±0.8	321.5±4.4
2 wt.% CG/ery	–	114.7±1.0	315.6±1.7
5 wt.% CG/ery	–	109.8±0.4	312.0±7.9
10 wt.% CG/ery	–	107.0±1.3	279.1±1.1
15 wt.% CG/ery	–	104.7±2.2	259.3±15.5
1 wt.% GG/ery	–	117.9±0.1	301.4±4.3
2 wt.% GG/ery	–	117.6±1.8	299.5±1.4
5 wt.% GG/ery	–	115.9±3.6	268.6±1.5
10 wt.% GG/ery	–	114.4±4.8	253.7±0.4
1 wt.% XG/ery	–	117.2±1.5	289.9±1.3
2 wt.% XG/ery	–	116.7±1.5	283.1±3.9

Through the non-isothermal DSC tests, it can be observed that the addition of thickeners has a decreasing effect on the melting point and the latent heat of fusion. CG has the most significant effect on reducing the melting point of erythritol, as 10 wt.% of CG lowers the melting point of erythritol by about 10.2°C, while 10 wt.% of GG only lowers it by 3.7°C. However, 10 wt.% of GG reduces the latent heat of fusion of erythritol by 24.3%, while CG at the same loading only reduces it by 16.8%. In addition, XG shows the most significant reduction in the latent heat of fusion of erythritol. For example, only 2 wt.% of XG decreases the latent heat of fusion of erythritol by 15.5%.

2.2. Degree of supercooling and crystallization rate

The supercooling behavior of GG- and XG-thickened erythritol was investigated by isothermal cooling test at a constant temperature from 10°C to 60°C at an increment of 10°C. In addition, for comparing the effects on supercooling behavior of erythritol between CG and GG, XG, we investigated the cooling process of CG-thickened erythritol at the same temperature of GG- and XG-thickened erythritol. During the preparation process, there are difficulties in preparing highly concentrated XG-thickened erythritol due to the high viscosity growth. In this study, the highest addition level of XG was only 2 wt.%. Therefore, as shown in Supplementary Figure 6, we compared the supercooling behavior of erythritol at different temperatures when CG, GG, and XG were added at a constant loading of 2 wt.%.

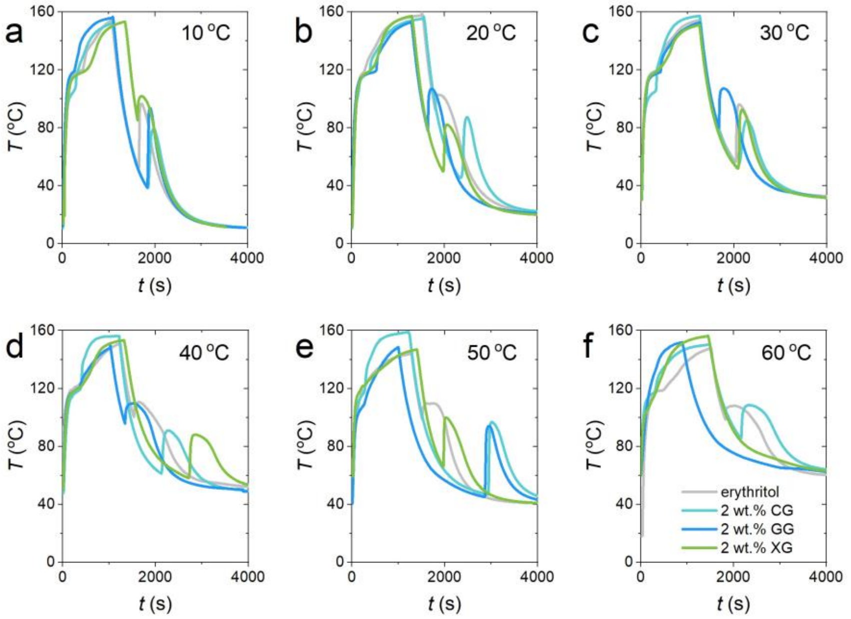

Supplementary Figure 6. The *T*-history curves of erythritol thickened by 2 wt.% CG, GG, and XG cooled at various temperatures: **a** 10°C, **b** 20°C, **c** 30°C, **d** 40°C, **e** 50°C, and **f** 60°C.

At the different isothermal cooling processes, CG consistently increases the degree of supercooling of erythritol. Although GG and XG also have similar thickening effects to that of CG, there are instances when they even decrease the degree of supercooling. For instance, at 10°C, XG reduces the degree of supercooling of erythritol from around 70°C to 23°C, as shown in Supplementary Figure 6a, and at 30°C, GG reduces it from around 60°C to 26.5°C, as shown in Supplementary Figure 6c. The phenomenon of decreasing the degree of supercooling of

erythritol was caused by the thermal decomposition products of thickeners. The addition of thickeners during the heating and melting process can result in thermal decomposition. Some decomposition products acted as nucleating agents during the cooling process, occasionally leading to a lowered degree of supercooling. In general, at low addition levels, all three gum-type thickeners have a significant effect in increasing the degree of supercooling, with CG showing the best performance. However, at low addition levels, none of the three thickeners can achieve an ultrastable supercooling behavior at extremely low temperatures.

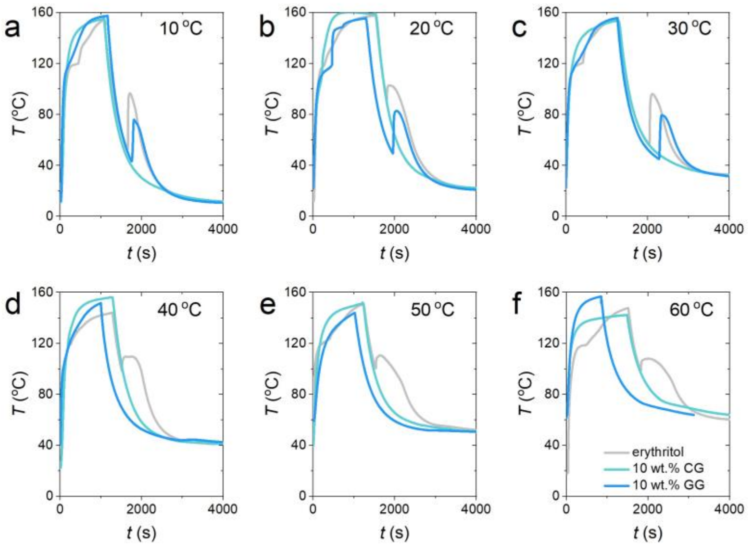

Supplementary Figure 7. The *T*-history curves of the erythritol thickened by 10 wt.% CG and GG cooled at various temperatures: **a** 10°C, **b** 20°C, **c** 30°C, **d** 40°C, **e** 50°C, and **f** 60°C.

At a high addition level of 10 wt.%, both CG and GG showed a stronger effect in increasing the supercooling degree of erythritol. In the isothermal cooling processes at various temperatures, no crystallization heat release phenomenon is observed at the 10 wt.% loading of CG, as shown in Supplementary Figure 7. However, for GG-thickened erythritol with a 10 wt.% addition level, although there is a significant enhancement in the supercooling behavior of erythritol, it exhibits crystallization heat release at lower cooling temperatures. As shown in Supplementary Figure 7a, b, c, at 10°C, 20°C, and 30°C, the addition of GG increases the degree of supercooling of erythritol to 74.8°C, 68.5°C, and 73°C, respectively, as compared to the initial values of 66.3°C, 26.5°C, and 60.5°C, respectively. GG can only stably prevent erythritol from crystallizing when the cooling temperature is above 40°C.

Through the comparison, it was found that CG shows the best performance on improving the supercooling behavior of erythritol among these three gum-type thickeners. Additionally, as shown in Supplementary Table 3, CG causes the least reduction in the latent heat of fusion of erythritol. Therefore, in the subsequent research, only CG was chosen for further investigation.

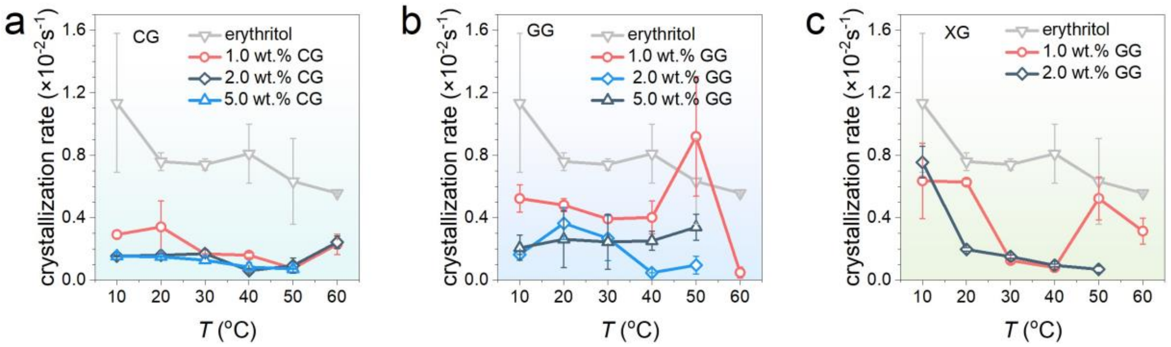

Supplementary Figure 8 The crystallization rate of erythritol with the three gum-type thickeners: (a) CG, (b) GG, and (c) XG.

Due to the increase in viscosity, the food thickeners not only increase the degree of supercooling of erythritol, but also reduces its crystallization rate during the discharging process. In this work, the point where the temperature starts to rise during the cooling process was identified as the onset of the crystallization process and the turning point of the temperature curve when it drops again indicating the completion of the crystallization process in the *T*-history curves[4,5]. Since the total mass used for each test sample is the same, the reciprocal of the time taken for the crystallization process was used to represent the crystallization rate. As shown in Supplementary Figure 8, the crystallization rate of pure erythritol gradually decreases with increasing the cooling temperature. This is because the decrease in the difference between the ambient temperature and the melting point results in a decrease in the driving force for crystallization, leading to a slower growth rate of the crystals[6].

It is evident that the addition of thickeners significantly reduces the crystallization rate of erythritol. Taking the 2 wt.% addition as an example, at cooling temperatures of 10, 20, 30, 40, and 50°C, CG reduces the crystallization rate by 85.3%, 92.5%, 77.0%, 79.1%, and 86.4%, respectively, GG reduces the crystallization rate by 53.9%, 36.8%, 38.8%, 50.5%, and 45.3%,

respectively, and XG reduces the crystallization rate by 33.3%, 74.2%, 76.4%, 88.3%, and 89.0%, respectively. Among these three thickeners, it can be observed that CG has the most significant effect in reducing the crystallization rate.

Although XG has a weaker enhancing effect on the supercooling behavior of erythritol compared to GG, it has a significantly stronger impact on reducing the crystallization rate. This is because XG has a better performance on increasing the viscosity, and the results related to this will be discussed in SI 2.3. A high viscosity will deteriorate natural convection in subcooled PCM during crystallization as indicated by the definition of Grashof (*Gr*) number that is given by

$$Gr = \frac{g\beta\rho^2\Delta TL^3}{\mu^2} \tag{S2-1}$$

where g is the gravitational acceleration (9.8 m·s^{-2}), β is the thermal expansion coefficient (K^{-1}), ρ is the density (kg·m^{-3}), ΔT is the typical temperature difference (K), L is the characteristic length (m), and μ is the dynamic viscosity (Pa·s). A higher viscosity, which is in the denominator of Eq. (S2-1), leads to a lower intensity of natural convection that will seriously slow down the crystallization rate.

More importantly, in terms of crystallization kinetics, the crystallization of the 10 wt.% GG-thickened erythritol in the supercooled liquid state would also be significantly suppressed by a high viscosity during heat retrieval, especially for those suffering from serious supercooling during cool-down[7]. The high viscosity reduces the molecule mobility and hinders the rearrangement of crystal lattices in supercooled thickened erythritol, thus leading to a decrease in the crystal growth rate, as determined by[8,9]

$$Y = \frac{fkT}{3\pi\mu a^2}\left[1-\exp\left(\frac{-\Delta G_c}{RT}\right)\right] \tag{S2-2}$$

where Y is the crystal growth rate (m·s), f is the fraction of sites on the crystal surface available for attachment, k is the Boltzmann constant (J·K), T is the absolute temperature for cooling (K), a is the thickness per molecular layer (m), ΔG_c is the free energy change between the liquid erythritol at melting point and solid erythritol at the temperature of T (J·mol^{-1}), and R is

the ideal gas constant (J·mol^{-1}·K^{-1}). The presence of viscosity in the denominator clearly indicates that the CG-thickened erythritol that is much more viscous slows down the crystal growth process.

2.3. Viscosity growth and rheological behavior

As shown in Supplementary Figure 9a, the viscosity of erythritol exhibits significant variations at low shear rates, rapidly decreasing with increasing the shear rate, and ultimately displaying expected Newtonian fluid characteristics at high shear rates. Remarkably, as shown in Supplementary Figure 9b and 7c, even with only 1 wt.% and 2 wt.% CG addition, the CG-thickened erythritol is still observed to exhibit its Newtonian fluid properties at high shear rates.

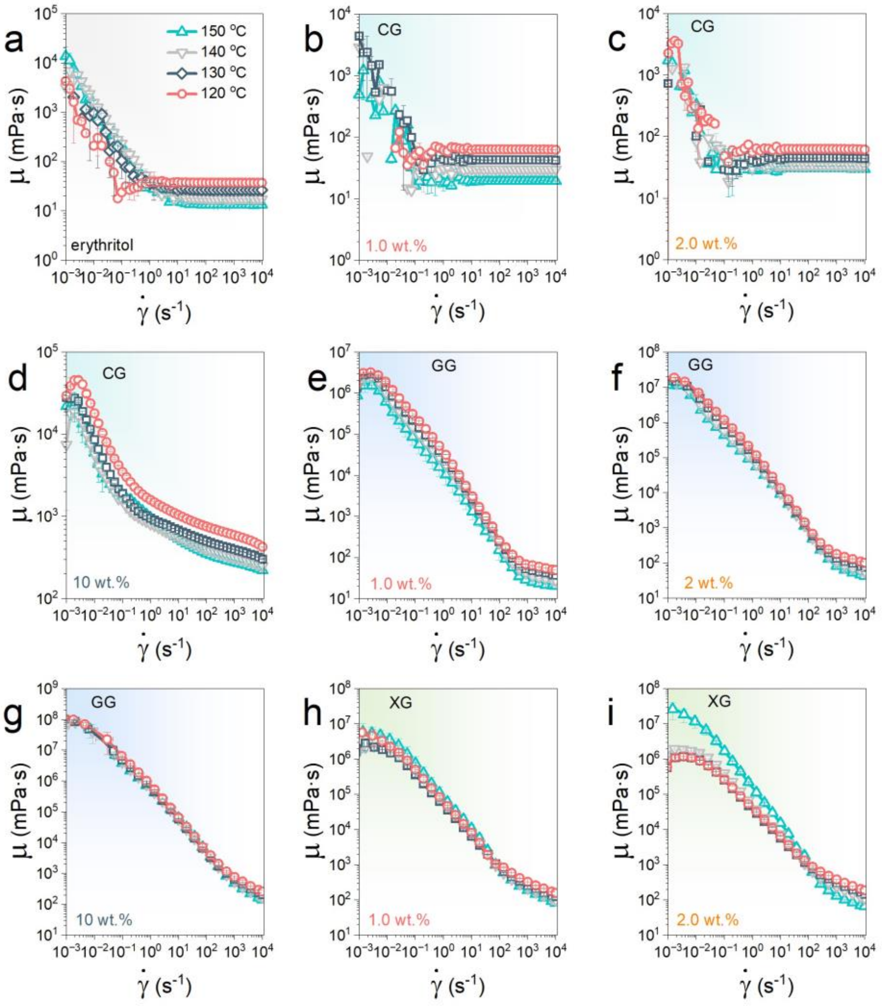

Supplementary Figure 9. The dynamic viscosity of erythritol as a function of shear rate with different thickeners. **a** pure erythritol, **b** 1.0 wt.% CG, **c** 2.0 wt.% CG, **d** 10 wt.% CG, **e** 1.0 wt.% GG, **f** 2.0 wt.% GG, **g** 10 wt.% GG, **h** 1.0 wt.% XG, and **i** 2.0 wt.% XG.

In contrast, when the same low mass fraction of GG and XG was added, the thickened erythritol shows non-Newtonian fluid behavior, with the value of viscosity dependent on the shear rate, as shown in Supplementary Figure 9e through Supplementary Figure 8i. In addition, with increasing the CG loading, the viscosity is improved significantly, leading to a gradual transition in the rheological behavior from Newtonian to non-Newtonian, as shown in Supplementary Figure 9d.

Furthermore, compared to the minimal improvement in viscosity of erythritol with a low amount of CG, both GG and XG have much more remarkable thickening effect. Supplementary Figure 10a demonstrates that at a shear rate of 1 s^{-1}, a 1 wt.% loading of CG just increases the viscosity of erythritol by 60.3% to 62.0 mPa·s at 120 °C, whereas GG and XG increases it by 1,054.2 and 1,599.2 times, respectively. As shown in Supplementary Figure 10b, at a high shear rate of 10,000 s^{-1}, CG increases the viscosity of erythritol by 57.2% to 58.5 mPa·s at 120 °C, while the improvement in viscosity with GG and XG decrease significantly, only increasing by 33.8% and 329%, respectively. Particularly with GG, its viscosity value at high shear rates is unexpectedly lower than that of CG. This is because non-Newtonian fluids tend to exhibit shear-thinning as increasing the shear rate. This phenomenon occurs when the CG concentration reaches 10 wt.%, as shown in Supplementary Figure 9g.

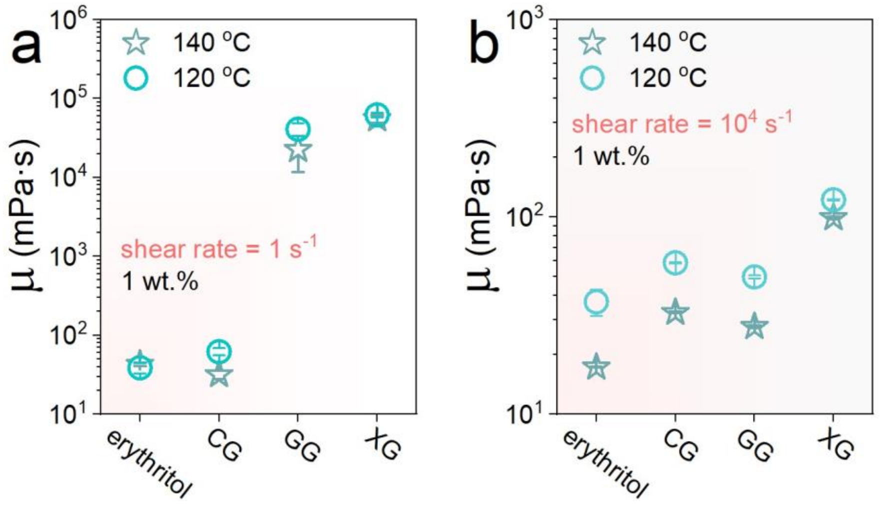

Supplementary Figure 10. The dynamic viscosity of erythritol with different thickeners at the shear rate of **a** 1 s^{-1}, and **b** 10,000 s^{-1}.

2.4. Summary of the comparison results

In summary, the addition of thickeners results in a decrease in the latent heat of fusion and melting point of erythritol to some extent. However, among thickeners with the same mass fraction, CG causes the least decrease in these of erythritol, and it exhibits the most significant and stable improvement in the degree of supercooling of erythritol. Although both GG and XG show a noticeable increase in viscosity at low shear rates, their effects are not as pronounced as that of CG with the same loading at high shear rates.

Additionally, due to their poor thermal stability, GG and XG are easy to undergo oxidation and decomposition during the heating process, and the byproducts generated act as nucleating agents, leading to an unstable effect on the degree of supercooling of erythritol. Considering the overall enhancement of erythritol's performance for seasonal solar energy storage, CG is selected as the most suitable thickener for further testing and investigation.

SI 3. Determination on the interfacial energy of the CG-thickened erythritol

3.1. Measurement of the solid-liquid contact angles by sessile drop method

The optical goniometer used for contact angle measurement is shown in Supplementary Figure11.

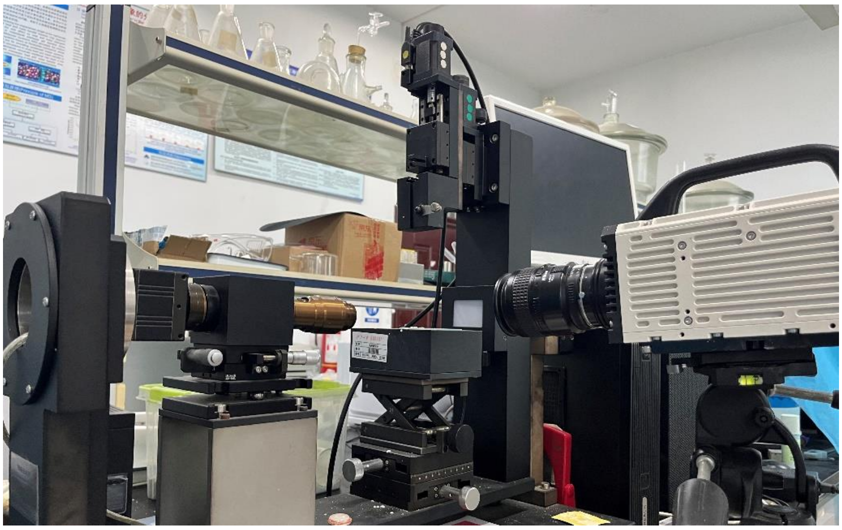

Supplementary Figure 11. The contact angle measuring device.

3.2. Measurement of the interfacial energy by pendant drop method

The pendant drop images taken for calculating the interfacial energy are shown in Supplementary Figure 12.

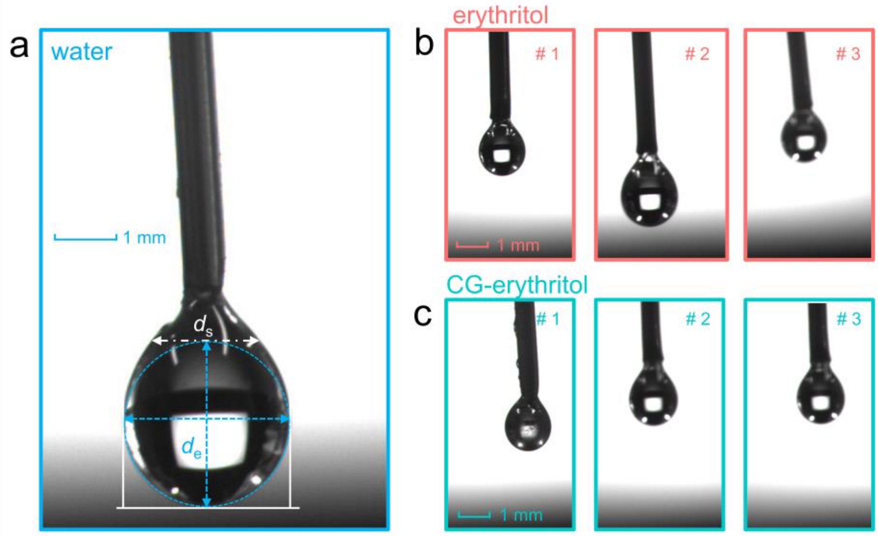

Supplementary Figure 12. The images of pendant drops for measuring the surface tension. **a** water, **b** erythritol, and **c** 15 wt.% CG-thickened erythritol.

Listed below (in Supplementary Table 4) are the calculated interfacial energy values for the three parallel tests of erythritol and the 15 wt.% CG-thickened erythritol samples.

Supplementary Table 4. The calculation results of interfacial energy.

	d_s (mm)	d_e (mm)	S	$1/H$	$\Delta\rho$ (kg·m^{-3})	γ_{lg} (mJ·m^{-2})
Erythrtiol-1	1.408	1.96	0.7166	0.7557	1338.71	36.27
Erythrtiol-2	1.297	1.83	0.7096	0.7753	1338.71	32.20
Erythrtiol-3	0.969	1.57	0.6166	1.1208	1338.71	34.45
CG-erythritol-1	1.189	1.93	0.6171	1.1183	1250.70	53.30
CG-erythritol-2	1.102	1.84	0.5992	1.2081	1250.70	52.43
CG-erythritol-3	1.156	1.89	0.6119	1.1432	1250.70	52.31

3.3. Validation of the method by measuring water surface tension

To verify the reliability of the pendant drop method, we also measured the surface tension (i.e., the gas-liquid interfacial energy) of water at room temperature following the same test procedure. As shown in Supplementary Figure 12a, the shape factor S of water is 0.6212, the $1/H$ in the empirical formula is 1.099, and the density difference is 996.71 kg·m^{-3}. The calculated surface tension value of water is 71.537 mJ·m^{-2}, in a very good agreement with the standard literature data (72.75 mJ·m^{-2}).

SI 4. Comparison of the stabilizing performance to other common thickeners

4.1. Phase change behaviors by isothermal and non-isothermal tests

As shown in Supplementary Figure 13, other common types of thickeners, including polyvinyl alcohol (PVA), carboxymethyl fiber (CMC) and sodium carboxymethyl cellulose (CMCNa) were also tested for their performance in stabilizing the supercooling behavior of erythritol.

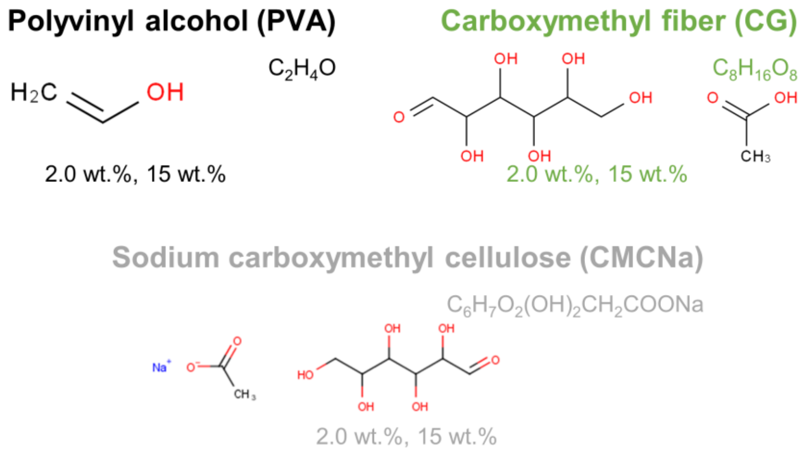

Supplementary Figure 13. The chemical structures of the other chemical synthetic types of thickeners tested.

As presented in Supplementary Figure 14, a comparative analysis is given on the impact of the various thickeners on the supercooling behavior of erythritol. In the non-isothermal tests by DSC, the addition of CMC, CMCNa, and CG, when the loading is >10 wt.%, all result in a significantly high degree of supercooling, except for PVA. The PVA-loaded samples (up to 15 wt.%) all can crystallize during the cooling process, as indicated by the small peaks on the exotherms. PVA is also known as a carcinogenic substance (by Toxic Substances Control Act), so it should not be considered as a potential additive for our seasonal PCM from the points of view of both the inefficient stabilization of supercooling and high health risk.

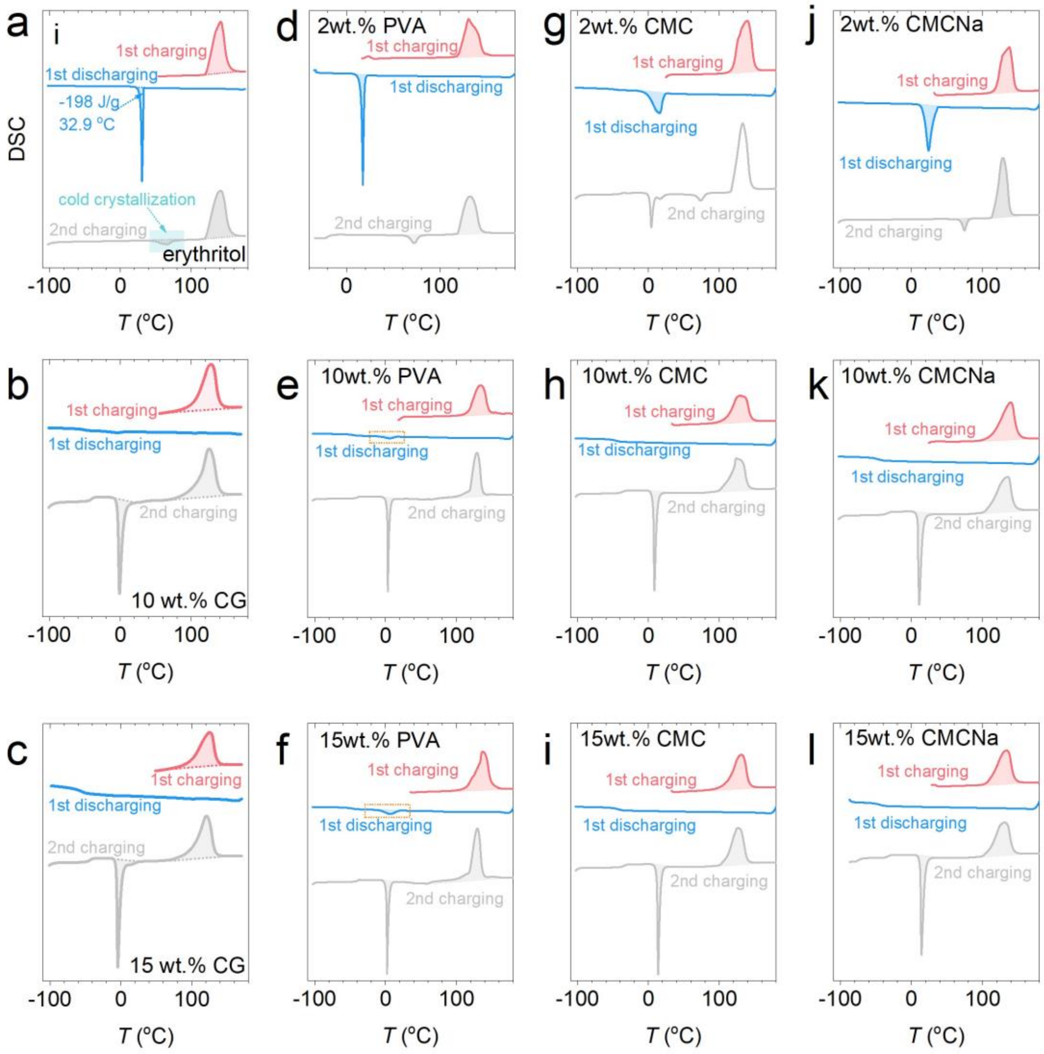

Supplementary Figure 14. The supercooling behavior and cold crystallization of erythritol thickened by various types of thickeners under non-isothermal test condition.

As shown in Supplementary Figure 15, subsequent isothermal test results show that at the constant high loading of 15 wt.%, the PVA-, CMC- and CMCNa-thickened erythritol are all unable to retain supercooled state at low temperatures below 0°C, resulting in rapid crystallization and discharging during the cooling process when exposed to a cold environment. While the erythritol near the cold tube wall promptly generates crystal nuclei and releases the heat, the portion close to the middle, i.e., near the *T*-thermocouple, has not yet been sufficiently cool. Such considerable temperature non-uniformity within the test tube results in the observation that the *T*-history curve of CMC-thickened erythritol reads a crystallization temperature of 97°C during the subsequent (second) cooling process.

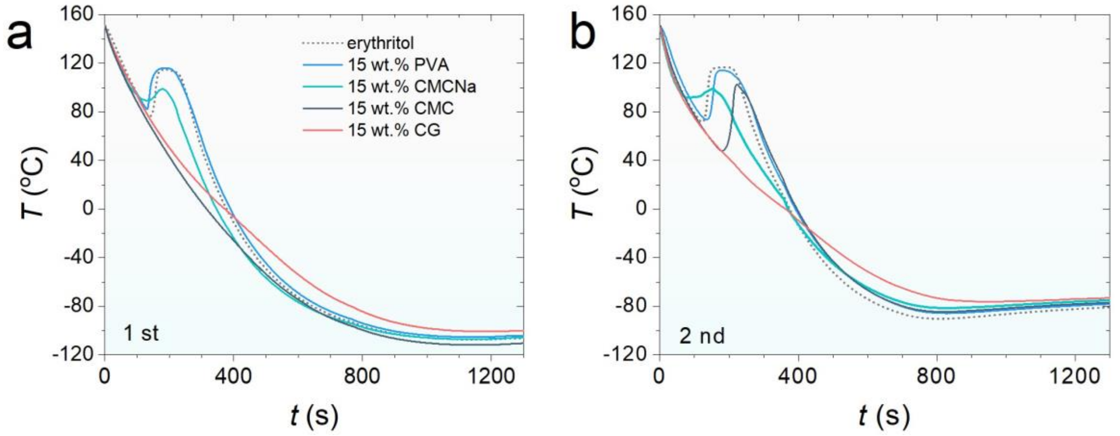

Supplementary Figure 15. The isothermal test results of erythritol samples with natural-type food thickeners and chemical synthetic types of thickeners.

4.2. Rheological behaviors

Due to the extremely outstanding thickening effect of CMC and CMCNa, it was difficult to measure the viscosity of the most concentrated 15 wt.% samples. Therefore, we compared the CMC- and CMCNa-loaded samples at a lower loading of 2 wt.% with that of CG.

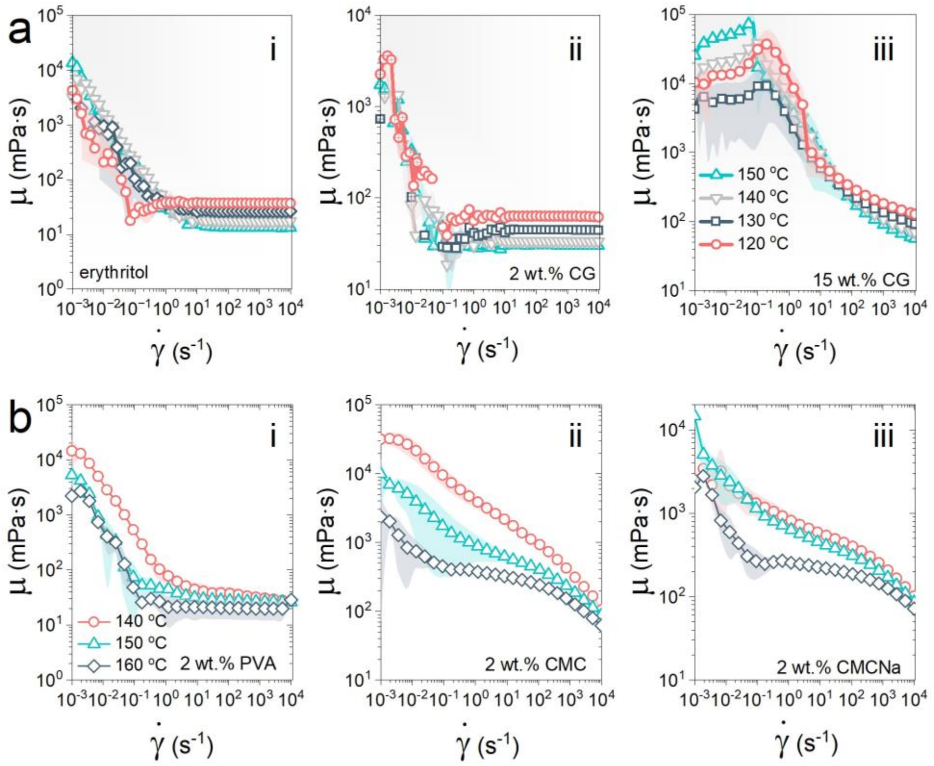

Supplementary Figure 16. The measured rheological behaviors of the thickened erythritol samples.

As shown in Supplementary Figure 16, it can be confirmed that both CMC and CMCNa exhibit much higher thickening effect than CG, while PVA shows a less remarkable thickening effect than CG, at the same 2 wt.% addition.

4.3. Interfacial energy

The contact angles of PVA-, CMC- and CMCNa-thickened erythritol were measured by the same sessile drop method, as presented in SI 3.1. The measurements were also done at the temperature of 80°C, and all the samples keep the same thickener loading of 2 wt.%. Here, we tested the contact angle of 2 wt.% CG-thickened erythritol for comparing with other thickened erythritol using chemical synthetic types of thickeners. As shown in Supplementary Figure 17, the contact angles of the PVA, CMC, and CMCNa-thickened erythritol are all much larger than that of pure erythritol (31.3°) and slightly greater than that of the CG-thickened erythritol (46.3°).

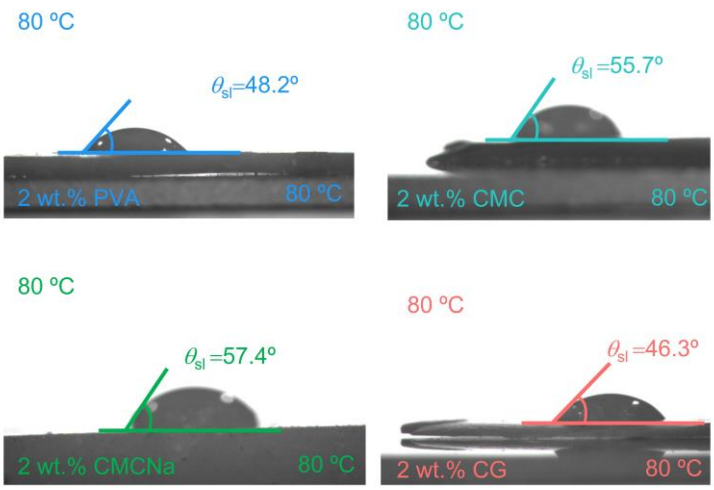

Supplementary Figure 17. The measured contact angles (θ_{sl}) of PVA-, CMC- and CMCNa-thickened erythritol.

Due to the high viscosity of the thickened erythritol samples, the liquid-gas interfacial energy was hard to be measured by the pendant drop method. Alternatively, we measured it at a temperature of 80°C by an automatic surface tensiometer (AFES, FST300M), as shown in Supplementary Figure 18a. Here we also tested the γ_{lg} of pure erythritol (32.6 ± 0.12 mJ·m^{-2}) by the tensiometer, and confirmed that the result is in good agreement to the that reported previously in SI 3.2 (34.31 ± 2.0 mJ·m^{-2}).

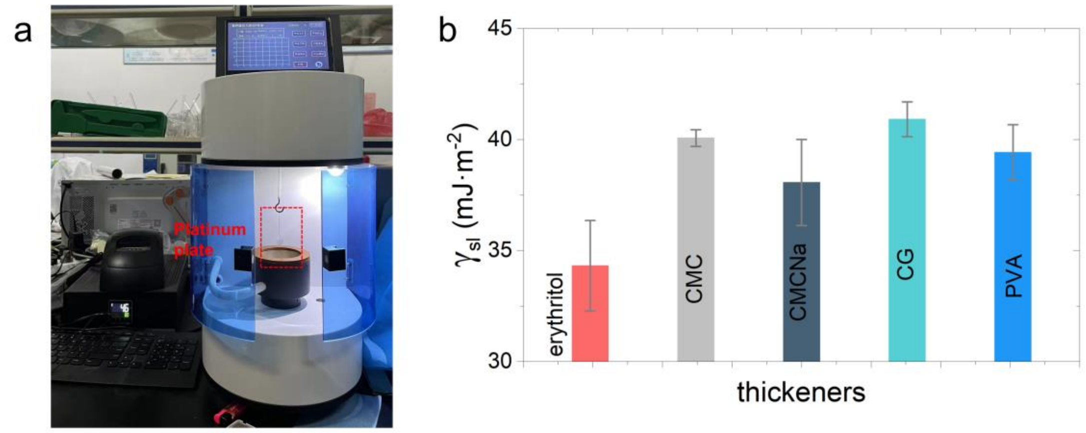

Supplementary Figure18. a The measurement of γ_{lg} using the automatic surface tensiometer, and **b** the measured results between pure erythritol and other thickened erythritol.

As shown in Supplementary Figure18 b, at the loading of 2 wt.%, although all of these thickeners can significantly increase the interfacial energy of erythritol, the relative increase by adding CG is deemed to be one of the best, with γ_{sl} = 42.5 ± 0.82 mJ·m^{-2} which is only slightly lower than that of CMC (43.1 ± 0.41 mJ·m^{-2}) and are fairly greater than those for PVA and CMCNa.

SI 5. Active triggering of crystallization of the CG-thickened erythritol by ultrasonication

5.1. Setup and procedure of the ultrasonication triggering test

After thickening the erythritol with 15 wt.% CG, the thickened erythritol sample was transferred to the ultrasonic device for triggering tests, as shown in the Supplementary Figure 19. The thermocouple and ultrasonic probe were inserted into the molten sample, and then the temperature of the sample was gradually cooled down to room temperature, forming the supercooled sample. After stabilizing at room temperature for a while, the crystallization and discharging process was triggered by activating the ultrasonication. And the power and duration of the ultrasonication applied were turned by the controller.

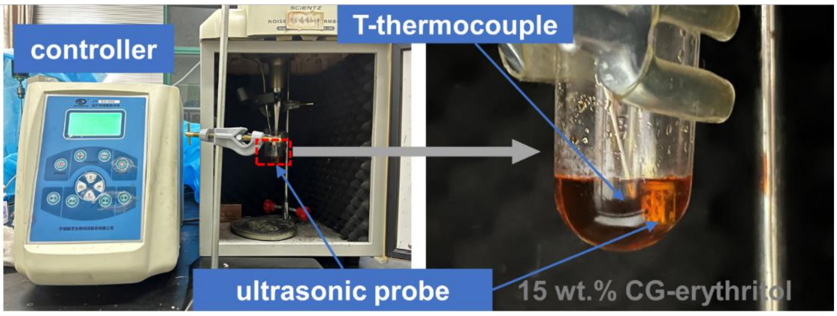

Supplementary Figure 19. The triggering test device by ultrasonication.

5.2. Effect of the ultrasonication power and duration on triggering effectiveness

The power and duration are two core factors dictating the efficacy of ultrasonic triggering. In our triggering tests, four different power levels (162.5 W, 325 W, 487.5 W, and 650 W) were compared at room temperature. We found that the two lower powers, i.e., 162.5 W and 325 W are not strong enough to trigger the crystallization of the highly-thickened erythritol samples. As increasing the power to 487.5 W, successful triggering was achieved. Seeming like a threshold power exists between 325 W and 487.5 W for triggering. Note that our goal was to verify if ultrasonication works for triggering of our new highly-supercooled PCM, so the exact threshold power was not pursuit.

We also showed that the successfulness of triggering is not influenced by the ratio of mass of the PCM to the ultrasonic power. As shown in Fig. 6(a-ii) in the main text and Supplementary Figure 20 below, only 10 g of the thickened erythritol sample remains non-crystallized at 325 W of ultrasonication, whereas the much greater amount of 200 g sample can be successfully triggered to crystallize at 487.5 W.

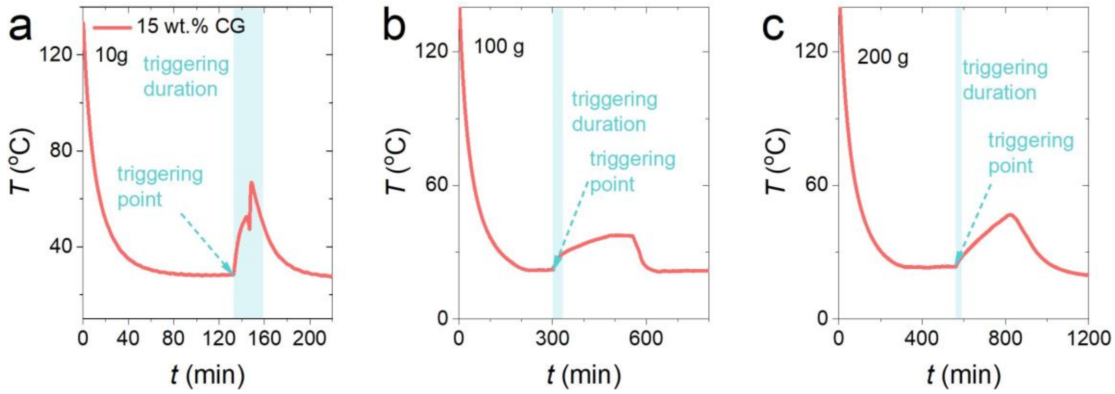

Supplementary Figure 20. Triggering crystallization of the 15 wt.% CG-thickened erythritol at various power-to-mass ratios.

Similar to the effect of ultrasonication power, the duration of ultrasonication for triggering also seems to have a threshold value. We found that when the duration is less than 10 min, even if the power of ultrasonication reaches 650 W, the thickened erythritol still cannot be triggered to crystallize. Only when the duration of ultrasonication exceeds 10 min, crystallization can be properly triggered.

5.3. Visualized observation of the crystallization process after triggering

In Supplementary Figure 21, a series of snapshots, with each time interval being 1 min, show the crystallization process of the 15 wt.% CG-thickened erythritol after triggered by ultrasonication for 10 min at the ultrasonic power of 487.5 W. Over the first 5 min of the ending of ultrasonication, the crystals start to grow from the ultrasonic probe, as indicated by the circles. Despite a heterogeneous surface extending into the supercooled liquid sample, however, there is no crystallization in the vicinity of the thermocouple. This observation suggests that the crystallization is not caused by the presence of a heterogeneous surface, thus confirming that the supercooled state of the 15 wt.% CG-thickened erythritol is very stable and that only the application of an ultrasonic power, instead of a cold heterogenous surface, can trigger the crystallization process.

During the continuous growth of the crystals, they always grow from the inside out, rather than starting from the lowest temperature point near the wall of the test tube. Until the crystallization process approaching the end, the supercooled liquid on the wall of the test tube gradually becomes part of the crystals.

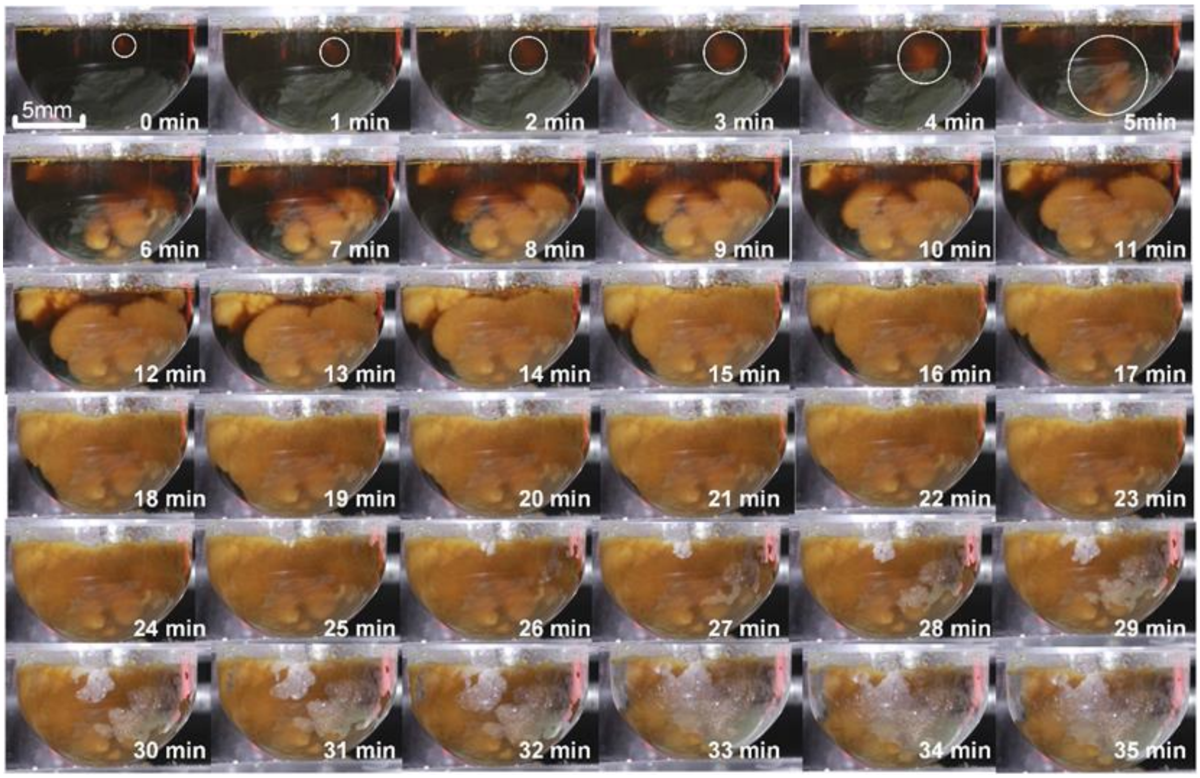

Supplementary Figure 21. The crystallization process of the 15 wt% CG-thickened erythritol as triggered by a relatively high ultrasonic power of 487.5 W for 10 min.

References

1. Shao, X.-F. *et al.* Screening of sugar alcohols and their binary eutectic mixtures as phase change materials for low-to-medium temperature latent heat storage. (I): Non-isothermal melting and crystallization behaviors. *Energy* **160**, 1078–1090 (2018).
2. Shao, X.-F. *et al.* Screening of sugar alcohols and their binary eutectic mixtures as phase change materials for low-to-medium temperature thermal energy storage. (II): Isothermal melting and crystallization behaviors. *Energy* **180**, 572–583 (2019).
3. Gunasekara, S. N., Chiu, J. N., Martin, V. & Hedström, P. The experimental phase diagram study of the binary polyols system erythritol-xylitol. *Sol. Energy Mater. Sol. Cells* **174**, 248–262 (2018).
4. Yuan, M. *et al.* Supercooling suppression and crystallization behaviour of erythritol/expanded graphite as form-stable phase change material. *Chem. Eng. J.* **413**, 127394 (2021).
5. Yang, S., Shao, X.-F., Shi, H.-Y., Luo, J.-H. & Fan, L.-W. Bubble-injection-enabled significant reduction of supercooling and controllable triggering of crystallization of erythritol for medium-temperature thermal energy storage. *Sol. Energy Mater. Sol. Cells* **236**, 111538 (2022).
6. Shao, X.-F., Chen, C.-L., Yang, Y.-J., Ku, X.-K. & Fan, L.-W. Rheological behaviors of sugar alcohols for low-to-medium temperature latent heat storage: Effects of temperature in both the molten and supercooled liquid states. *Sol. Energy Mater. Sol. Cells* **195**, 142–154 (2019).
7. Yuan, M., Cao, D., Liu, C., Xu, C. & Liao, Z. Temperature-dependent growth kinetic and crystallization behavior of the supercooled erythritol as a phase change material in long-term heat storage. *Chem. Eng. J.* **469**, 143743 (2023).
8. Diogo, H. P. & Moura Ramos, J. J. Slow molecular mobility in the crystalline and amorphous solid states of glucose as studied by thermally stimulated depolarization currents (TSDC). *Carbohydr. Res.* **343**, 2797–2803 (2008).
9. Turunen, K., Yazdani, M. R., Puupponen, S., Santasalo-Aarnio, A. & Seppälä, A. Cold-crystallizing erythritol-polyelectrolyte: Scaling up reliable long-term heat storage material. *Appl. Energy* **266**, 114890 (2020).

10. Ambwani, D. S. & Fort, T. Pendant Drop Technique for Measuring Liquid Boundary Tensions. in *Surface and Colloid Science* (eds. Good, R. J. & Stromberg, R. R.) 93–119 (Springer US, 1979).
